# The impact of cow genetics on the somatotropic axis in pasture-based systems: Implications for production and reproduction

**DOI:** 10.3168/jdsc.2025-0985

**Published:** 2026-03-20

**Authors:** S.T. Butler, E.M. Sitko, M.C. Lucy

**Affiliations:** 1 Teagasc, Animal and Grassland Research and Innovation Centre, Moorepark, Fermoy, Co. Cork, P61 P302 Ireland; 2 Department of Animal Science, University of Missouri, Columbia, MO 65201

## Abstract

Seasonal, pasture-based milk production imposes distinctive physiological demands on dairy cows: compact calving, prompt resumption of estrous cyclicity and completion of uterine involution, achieving peak milk production on a diet primarily composed of grazed grass with short-term variability in supply and quality, and re-establishing pregnancy during a short breeding period. Central to these adaptations is the somatotropic axis—growth hormone (GH), hepatic GH receptor (GHR), insulin-like growth factor-1 (IGF1), 6 IGF binding proteins (IGFBP1–IGFBP6), and the IGF binding protein acid labile subunit (IGFALS)—which orchestrates nutrient partitioning, lactation, and the return to cyclicity and potential to reestablish pregnancy. This review summarizes the insights from experiments conducted in Ireland and New Zealand comparing different cow genotypes managed under grazing systems (e.g., North American vs. New Zealand Holstein-Friesian cows; Holstein-Friesian cows with divergent fertility merit but similar milk merit) and integrates herd-scale postpartum phenotypes linked to reproduction. In seasonal-calving, pasture-based systems, breeding indexes to improve cow genetic merit (greater emphasis on fertility and milk solids) are associated with a more favorable somatotropic axis profile (greater serum IGF1 during lactation), more conservative nutrient partitioning (less BCS loss), and superior uterine health, estrous expression, luteal function, and conception, without compromising milk solids output per cow. The biology of the somatotropic axis in dairy cows has an important effect on nutrient partitioning and fertility under pasture-based systems of milk production.

## Highlights

• Cow genetics drive hormone signals that balance milk, body reserves, and fertility.
• High fertility index cows maintain higher IGF-1 during lactation.
• High fertility index cows lose less body condition after calving on pasture-based diets.
• Higher IGF-1 is linked with better uterine health, stronger estrus, and greater conception.

## Graphical Abstract

Seasonal, pasture-based dairy systems require cows to calve compactly, achieve peak milk output on variable grass diets, and become pregnant again within a short breeding season. This review explains how cow genetics influence the growth hormone-insulin-like growth factor-1 (IGF-1) system that helps direct nutrients toward milk, body reserves, and reproduction. Across studies in Ireland and New Zealand, cows bred with a greater emphasis on fertility and milk solids had more favorable hormone profiles during lactation, including higher circulating IGF-1 concentrations, earlier restoration of a “coupled” growth hormone-IGF axis after calving, and less loss of body condition. These animals also had better uterine recovery after calving, stronger expression of estrus, improved ovarian function, and higher conception, while maintaining milk solids output per cow. Overall, selecting cows to have better genetic merit for fertility traits can improve hormonal regulation that supports both productive performance and timely reestablishment of pregnancy in grazing herds. BCS = body condition score; GH = growth hormone.

**Figure:**
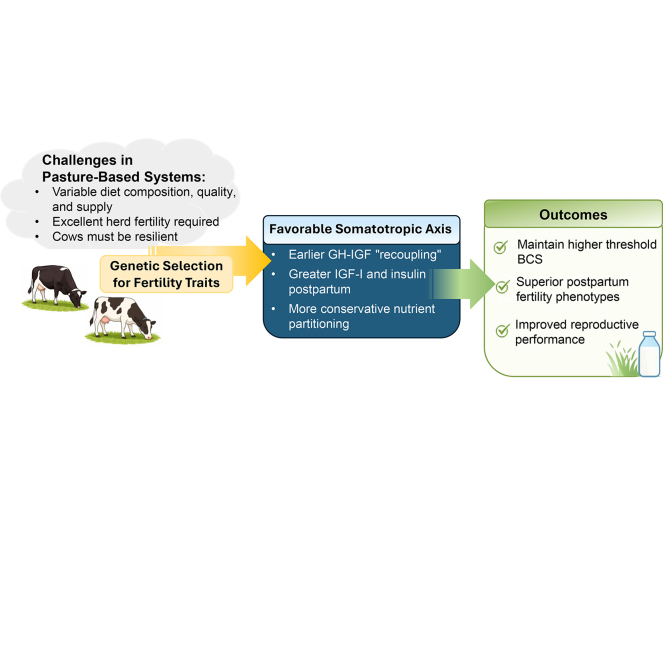

In pasture-based systems, the nutritional status of lactating cows is shaped largely by the farm stocking rate, annual pasture DM yield, planned supplementation strategy (concentrates, conserved forages), and management responses to fluctuations in weather conditions that reduce pasture growth (i.e., temperature, rainfall). In seasonal pasture-based systems, cows calve in late winter and can be exposed to a variable diet during early lactation, and the diet can change markedly between seasons, reflecting changes in pasture availability, pasture quality, and supplementation strategy. In addition, prevailing milk and concentrate feed prices can influence decisions related to concentrate supplementation. The purpose of this review is to examine the effect of genetic background on the regulation of the somatotropic axis in dairy cows managed under pasture-based systems and explore the implications for production and reproduction.

Dairy cow fertility has been described as “the ability of the animal to conceive and maintain a pregnancy if served at the appropriate time in relation to ovulation” (Darwash et al., 1997, p. 9). Evans et al. (2006) reported that across 14 Irish spring-calving herds, calving risk to first service fell from 55% in 1990 to 44% in 2001, coincident with continual genetic and phenotypic increases in milk production per cow. It became recognized that the endocrine and metabolic adjustments required to initiate and sustain lactation conflicted with the physiological conditions that support optimal fertility in high-producing dairy cows (Lucy, 2001). Genetic selection to increase milk output had inadvertently disrupted circulating concentrations of key hormones (e.g., IGF1, progesterone) and energy metabolites (e.g., glucose) that influence the reproductive axis (Buckley et al., 2000; Crooker et al., 2001). During the last quarter-century, it has become generally acknowledged that (1) breeding values for fertility traits declined substantially during the period when selection pressure was directed almost exclusively toward milk yield (Weigel, 2004); and (2) the prioritization of nutrient partitioning toward the mammary gland during the initiation of lactation can interfere with the biological processes required for successful conception and pregnancy establishment (Butler, 2013). Since that time, fertility traits have been included in the breeding objectives and selection indices in most countries, and recent trends indicate that measures of reproductive performance (pregnancy per artificial insemination, calving interval, and so on) are improving (Ma et al., 2019; Brito et al., 2021).

Reproduction in female cattle is strongly influenced by nutritional status. Age at puberty, postpartum interval to resumption of estrous cyclicity, and likelihood of pregnancy establishment following insemination are all strongly associated with a variety of measures that indicate nutritional status, including BCS, blood metabolic hormones, and blood energy metabolites (Buckley et al., 2003; Rojas Canadas et al., 2020b). Insulin-like growth factor-1 (IGF1) is a metabolic hormone and an important regulator of ovarian function. As plasma IGF1 concentrations are depressed during early lactation, this has been posited as a possible reason for impaired reproductive performance in some animals. The mechanisms leading to reduced circulating concentrations of IGF1 are complex, and a brief overview of the growth hormone (**GH**)-IGF axis is presented in the following paragraphs (Table 1). Most of the research that has elucidated the regulation of the GH-IGF axis was conducted on laboratory animal species and on high-producing cows managed under confinement TMR management systems.

**Table 1:** Key components of the somatotropic (GH-IGF) axis in dairy cattle, expected changes in response to negative energy balance (NEB), primary mechanisms linking energy status to the somatotropic axis, and general effects of genetic selection and strain differences observed in pasture-based systems^1^

Component	Source or principal tissue	Primary function or action	Response to NEB	Mechanism linking nutrition to axis	Response to genetic selection (Fert+ and NZ cows)
GH	Anterior pituitary	• Promotes lipolysis and gluconeogenesis; supports lactation • Stimulates hepatic IGF1 mRNA expression	↑	GH-resistant state (axis uncoupled) when serum GH increases but does not stimulate hepatic IGF1 synthesis	• Shorter period of GH resistance • Earlier recoupling of somatotropic axis
GH receptor	Liver has greatest abundance; GHR1A mRNA is liver-specific	• Mediates hepatic response to GH • Hepatic GHR abundance determines liver IGF1 output	↓	Hypoinsulinemia reduces hepatic GHR1A mRNA, causing GH resistance	↑ GHR1A mRNA in early lactation
IGF1	Liver is the main source of IGF1 in circulation	• Anabolic hormone • Mediates many GH effects	↓	Reduced hepatic IGF1 expression during GH resistance and hypoinsulinemia	↑ Serum IGF1 postpartum
Low-MW IGFBP (IGFBP-2, 4, 5, 6)	Liver is major source	• Bind IGF1, reduce free IGF1 fraction, and alter tissue delivery • Short IGF1 t1/2 (30–90 min)	↑	Hypoinsulinemia increases serum concentrations of low-MW IGFBP	↓ Hepatic expression of low-MW IGFBP postpartum
IGFBP3 and IGFALS	Liver is major source	• Bind IGF1, reduce free IGF1 fraction, and alter tissue delivery • Long IGF1 t1/2 in ternary complex (˜12–15 h)	↓	Hypoinsulinemia decreases serum concentration of IGFBP-3	—
Insulin	Pancreas	• Promotes nutrient storage • Upregulates hepatic expression of GHR1A and IGF1	↓	• Hypoinsulinemia reduces hepatic GHR1A and IGF1 mRNA. • Hypoinsulinemia shifts IGF1 from circulating in ternary complex to circulating in low-MW IGFBP	↑ Serum insulin postpartum

1 GH = growth hormone; GHR = GH receptor; IGF1 = insulin-like growth factor-1; IGFBP = insulin-like growth factor binding protein; IGFALS = IGF binding protein acid labile subunit; t1/2 = half-life.

Circulating IGF1 is produced primarily by the liver in response to GH (Jones and Clemmons, 1995). Hepatic expression of IGF1 is also acutely responsive to nutritional status (Pell et al., 1993). The stimulatory effect of GH on liver IGF1 synthesis is “uncoupled” during periods of nutritional stress or negative energy balance and is “recoupled” when these conditions improve (Breier et al., 1986; McGuire et al., 1995a). During this time the liver becomes refractory to GH (Vicini et al., 1991), and circulating IGF1 concentrations are markedly reduced. The inability of GH to stimulate hepatic IGF1 production during periods of negative energy balance (**NEB**) is termed GH resistance and has been documented in many species. Hepatic GH receptor (GHR) abundance is positively correlated with plasma IGF1 and nutritional status, and thus the abundance of liver GHR is a key regulatory mechanism for determining activity within the GH-IGF axis (Breier, 1999).

GHR belongs to the large family of cytokine receptors, and it is through this receptor that the direct and indirect actions of GH are mediated. The effects of GH are primarily dependent on 2 factors: (1) the number of GH receptors, and (2) the activity of the GHR second messenger system (Carter-Su and Smit, 1998). In cattle, the highest levels of GHR expression are found in the liver, with detectable levels also expressed in muscle, fat, the kidneys, the heart, and tissues of the reproductive tract (Lucy et al., 1998). In cattle, GHR mRNA is produced as at least nine 5′-untranslated region (**5′-UTR**) variants named GHR 5′-UTR 1A to 1I (Jiang and Lucy, 2001). The major 5′-UTR variants are 1A, 1B, and 1C, with 1A and 1B being highly expressed in the liver and 1B and 1C being highly expressed in muscle. The promoter (P1) that drives the expression of GHR 1A is liver-specific and is likely the principal determinant of liver GHR concentrations (Kobayashi et al., 1999a). It is regulated developmentally and nutritionally, and is responsive to exogenous GH administration (Lucy et al., 1998; Kobayashi et al., 1999b). Hepatic expression of GHR 1A mRNA is greater in late lactation than in early lactation (Kobayashi et al., 1999a), reflecting the effect of energy status on transcript abundance; indeed, the decline in total GHR mRNA on the day of parturition is due to specific downregulation of GHR 1A. Binding of GH leads to receptor dimerization, followed by phosphorylation and activation of intracellular tyrosine kinases (Carter-Su and Smit, 1998). Like many of the receptors in the cytokine family, GHR uses the JAK-STAT signal transduction pathway (Kelly et al., 1994). The activated GHR recruits and activates the cytoplasmic tyrosine kinase JAK2 (Janus kinase 2), stimulating the activation of signaling molecules, including members of the family of signal transducers and activators of transcription (STAT), which then dimerize and bind to specific DNA sequences in the nucleus and regulate a variety of GH-dependent genes (Carter-Su et al., 2000).

Circulating insulin concentrations decline during the period of transition from gestation (positive energy balance) to lactation (negative energy balance), and then gradually increase again during lactation as energy balance returns to positive values. The responsiveness of adipose and muscle tissues to insulin is reduced during lactation (Vernon and Sasaki, 1991), allowing a greater quantity of nutrients to be diverted to the mammary gland. Although this is often referred to as insulin resistance, only some of the insulin-responsive processes are impaired (Wilson et al., 1996). During the passage from pregnancy to lactation, the changes in plasma insulin and IGF1 are parallel, and the profile of plasma GH is reversed. The diminished influence of GH on hepatic IGF1 output during early lactation is reflective of energy status. In animals that are in positive energy balance, treatment with GH has profound effects on carbohydrate metabolism, including increased hepatic gluconeogenesis, reduced glucose utilization by peripheral tissues, and decreased insulin sensitivity (Etherton and Bauman, 1998). Of note, after administration of exogenous GH, an increase in circulating insulin occurred in parallel with an increase in plasma IGF1, but only in adequately fed cows; in underfed cows, an insulin response was not observed, and plasma IGF1 was not increased (Vicini et al., 1991; McGuire et al., 1995a).

A chronic elevation in circulating insulin (via hyperinsulinemic-euglycemic clamp) increases plasma IGF1 in mid-lactation cows (i.e., positive energy balance; McGuire et al., 1995b) and in cows during early lactation (i.e., negative energy balance; Butler et al., 2003; Rhoads et al., 2004). Insulin regulates plasma IGF1 concentration by increasing hepatic mRNA abundance of both GHR 1A and IGF1 (Butler et al., 2003). In circulation, IGF1 is bound by IGF-binding proteins (IGFBP), and 6 IGFBP with variable molecular weights (**MW**) have been identified in cattle. The half-life of IGF1 varies dramatically depending on its circulating form: ~10 min as free peptide, 30 to 90 min when bound to a low-MW IGF-binding protein (IGFBP2, IGFBP4, IGFBP5, or IGFBP6), and 12 to 15 h when incorporated into the ternary complex with IGFBP3 and acid-labile subunit (Jones and Clemmons 1995). Insulin administration shifted the circulating IGFBP profile by decreasing the plasma concentration of the smaller MW binding protein complexes (especially IGFBP-2, the most abundant binding protein) and increasing the concentration of the greater-MW IGFBP-3 (Butler et al., 2003). Hence, increasing the proportion of IGF1 associated with IGFBP-3 slows the overall clearance of IGF1 from circulation (Jones and Clemmons 1995) and provides another mechanism for energy status (via insulin) to regulate the concentration of circulating IGF1.

In the following paragraphs, the effects of cow genetic background and nutritional management strategy on nutrient partitioning and the GH-IGF axis in pasture-based systems will be summarized.

In pasture-based systems, achieving low-cost milk output relies heavily on grazed pasture as the primary feed source, which in turn necessitates excellent herd fertility so that calving is concentrated around the period of spring pasture growth onset. Several comparative studies have evaluated lactating cows with North American (**NA**) and New Zealand (**NZ**) genetic backgrounds (Harris and Kolver, 2001; Horan et al., 2004, 2005a,b; McCarthy et al. 2007b). The objective of these studies was to compare production, reproduction, and farm profitability between cows selected for confinement TMR systems (NA) and cows selected for pasture-based grazing systems (NZ) when both strains were managed under diverse pasture-based systems of production. When the groups of animals used in these studies were assembled, the NA Holstein strain of cows had been selected largely for greater milk yield, larger body size, and increased angularity within systems characterized by year-round calving and substantial concentrate supplementation, with limited emphasis on fertility traits. In contrast, selection pressure in the NZ Holstein-Friesian population focused on milk solids yield (fat plus protein) together with enhanced fertility and longevity within a predominantly pasture-based production environment (Horan et al., 2005a). Collectively, these comparisons revealed that NZ Holstein-Friesians produced less milk volume but similar quantities of milk solids, maintained greater BCS, had lighter body weight, and had better reproductive performance compared with the NA Holsteins (Harris and Kolver, 2001; Horan et al., 2004, 2005b). Greater longevity in the NZ strain also translated into greater profitability under pasture-based systems (McCarthy et al., 2007b).

The physiological basis for the superior fertility observed in the NZ strain has been partially elucidated. Extensive evidence links postpartum BCS loss and BCS at breeding with improved phenotypic fertility (Buckley et al., 2003; Roche et al., 2009). Compared with NA cows, NZ animals maintained a higher baseline BCS throughout the gestation–lactation cycle, mobilized less condition after calving (though the initial rate of mobilization was comparable), and diverted a greater proportion of nutrients toward restoring body reserves during mid- and late lactation (Roche et al., 2006; McCarthy et al., 2007a; Patton et al., 2008). Despite these differences, calculated energy balance during the first 20 wk of lactation was similar in both strains (Patton et al., 2008). Glucose tolerance tests revealed faster glucose clearance in NZ cows during both early (Chagas et al., 2009) and mid-lactation (Patton et al., 2009), implying greater insulin resistance in NA cows and a stronger drive to prioritize nutrient allocation to the mammary gland at the cost of maintaining or replenishing BCS.

During early lactation, coordinated endocrine and metabolic adjustments are required to support milk synthesis. Most cows experience at least some level of NEB during this period, resulting in mobilization of body reserves and an associated uncoupling of the somatotropic axis. Circulating concentrations of IGF1 were greater in NZ cows compared with NA cows (Patton et al., 2008; Lucy et al., 2009). Research undertaken in New Zealand by Lucy et al. (2009) examined endocrine profiles and hepatic gene expression in NZ and NA cows; the NZ cows maintained a more “coupled” somatotropic axis during early lactation, characterized by lower circulating GH, greater IGF1, and increased hepatic GHR1A expression, which supported less BCS mobilization. In contrast, NA cows exhibited an “uncoupled” axis accompanied by greater BCS loss (Lucy et al., 2009). Notably, that study did not detect strain differences in hepatic IGF1 gene expression. A similar study conducted in Ireland did not detect differences in hepatic GHR1A expression, but NZ cows had greater hepatic IGF1 mRNA abundance and changes in IGF-binding protein expression that would favor a longer IGF1 half-life in circulation (McCarthy et al., 2009). Taken together, these studies imply that adaptation to the physiological challenges of early lactation was not tolerated as well by the NA strain compared with the NZ strain when managed under similar pasture-based systems. This likely reflects NA cows having greater genetic potential for milk production but being unable to meet that potential on pasture-only diets. In support of this, several pasture-based studies reported that the milk production response to additional concentrate supplement was greater in cows with better genetic merit for milk production (see review by Butler, 2014).

Cows with greater genetic merit for milk production have frequently been reported to have reduced reproductive performance compared with cows of average genetic merit (Lucy and Crooker, 1999; Buckley et al., 2000; Kennedy et al., 2003). Nonetheless, it is improbable that high phenotypic milk output is the direct cause of reduced fertility. Cows that differ in milk yield often also differ in a suite of related phenotypes (e.g., milk composition, BW, BCS, feed intake capacity), and hence it is difficult to pinpoint genetically driven pathways responsible for reproductive failure when working with contrasting phenotypic groups. To overcome these limitations, a lactating cow research model was established at Teagasc Moorepark to compare cows with similar genetic merit for milk production traits but with divergent genetic merit for fertility traits, classified as **Fert+** (good fertility) or **Fert−** (poor fertility). These animals did not differ in proportion of Holstein breed genetics, and had similar BW, milk yield, and milk constituents, but Fert− cows had inferior fertility compared with Fert+ cows (Cummins et al., 2012a). The findings generated using this model indicated many underlying causes of reduced fertility in Fert− animals. Of note, energy balance, nutrient partitioning, and many components of the GH-IGF axis were markedly different between the 2 genotypes.

Metabolic hormone profiles in Fert+ versus Fert− cows broadly reflect patterns previously described for NZ and NA strains, with similar implications for BCS. In early lactation, Fert+ cows have greater circulating insulin and glucose concentrations (Cummins et al., 2012a; Moore et al., 2014), as well as greater circulating IGF1 concentrations throughout lactation (Cummins et al., 2012a). Elevated glucose in the peripartum period has been associated with earlier ovulation (Butler et al., 2006) and increased conception rate (Garverick et al., 2013). The mechanistic basis for the differences in circulating IGF1 concentrations between Fert+ and Fert− cows was elucidated by (Cummins et al., 2012b). Although IGF1 concentrations were greater in Fert+ cows throughout lactation, hepatic IGF1 gene expression was elevated only from mid- to late lactation. During early lactation, when Fert+ cows also had greater circulating IGF1 concentrations, Fert+ cows had reduced hepatic expression of low-MW binding proteins, thereby facilitating a greater proportion of IGF1 to be bound within the long-lived ternary complex. Thus, genetic merit for fertility traits influences multiple components of the somatotropic axis: IGF1 synthesis, bioavailability, and stability.

Transcriptomic analyses of liver and muscle tissue collected from Fert+ and Fert− cows during late pregnancy, early lactation, and mid-lactation were reported by Moran et al. (2016). One of the biological themes apparent from the functional annotation of differentially expressed gene sets was “metabolism, lipid and carbohydrate”–related functions. This biological theme included the terms “gluconeogenesis” and “extracellular growth factor” in late pregnancy; “biosynthetic process,” “lipid lipoprotein,” and “metabolic process” in early lactation; and “lipid” and “lipoprotein particle” at mid-lactation. The transcriptomic profile provided evidence that key gene pathways related to metabolism, bioenergetic status, and nutrient partitioning (including IGF1) were differentially regulated, suggesting that metabolic processes are fundamental to the phenotypic divergence in fertility in this animal model (Moran et al., 2016). Consistent with their more favorable metabolic state, Fert+ cows maintained greater BCS throughout lactation and experienced less postpartum loss than Fert− cows. Their ability to sustain greater BCS was supported by greater DMI during early lactation (Moore et al., 2014) and required coordinated endocrine signaling to regulate nutrient partitioning across tissues.

A multi-year research study was conducted in Ireland to compare elite genetic merit cows (top 5% based on Economic Breeding Index) versus national average genetic merit cows, managed under different pasture-based feeding treatments (O'Sullivan et al., 2020). The elite cows maintained greater serum IGF1 concentrations and greater BCS throughout lactation, and had better phenotypic reproductive performance and greater survival to fifth lactation. Rojas Canadas et al. (2020a) examined the association between genetic merit for fertility traits and postpartum fertility phenotypes in grazing dairy herds located in the Munster region of Ireland (n = ~2,600 cows in 35 herds; study cows were parity 1 and parity 2). Individual cow data captured at wk 3 and 7 postpartum indicated that cows with better genetic merit for fertility traits were more likely to have target BCS (2.75 to 3.25 using a scale from 1 to 5), to have more favorable metabolic status (based on blood concentrations of glucose, BHB, and nonesterified fatty acid), to have a corpus luteum present on the ovary (indicator of earlier resumption of estrous cyclicity), and to have better uterine health status compared with cows that had poor genetic merit for fertility traits, and these phenotypes were subsequently associated with better reproductive performance during the breeding season (Rojas Canadas et al., 2020a,b).

In conclusion, selection for milk solids and fertility traits (e.g., NZ strain, Fert+ cows, elite cows) results in cows with greater postpartum circulating concentrations of insulin and IGF1, faster recoupling of the GH-IGF axis, and conservative nutrient partitioning that preserves BCS and supports timely uterine recovery and cyclicity. Cow genetic background affects multiple components of the somatotropic axis in pasture-based systems, and this has important consequences for production and reproduction.
